# Safety, feasibility and complications during resective pediatric epilepsy surgery: a retrospective analysis

**DOI:** 10.1186/1471-2253-14-71

**Published:** 2014-08-18

**Authors:** Marcus O Thudium, Marec von Lehe, Caroline Wessling, Jan-Christoph Schoene-Bake, Martin Soehle

**Affiliations:** 1 Department of Anesthesiology and Intensive Care Medicine, Sigmund-Freud-Str. 25, 53105 Bonn, Germany; 2 Department of Pediatrics, University of Freiburg Medical Center, Heiliggeiststraße 1, 79106 Freiburg im Breisgau, Germany; 3 Department of Neurosurgery, University of Bochum, Knappschaftskrankenhaus, In der Schornau 23-25, 44892 Bochum, Germany; 4 Department of Neurosurgery, Sigmund-Freud-Str. 25, 53105 Bonn, Germany

**Keywords:** Epilepsy surgery, Neuroanesthesia, Pediatric neuroanesthesia, Pediatric neurosurgery

## Abstract

**Background:**

Resective epilepsy surgery is an established and effective method to reduce seizure burden in drug-resistant epilepsy. It was the objective of this study to assess intraoperative blood loss, transfusion requirements and the degree of hypothermia of pediatric epilepsy surgery in our center.

**Methods:**

Patients were identified by our epilepsy surgery database, and data were collected via retrospective chart review over the past 25 years. Patients up to the age of 6 years were included, and patients with insufficient data were excluded.

**Results:**

Forty-five patients with an age of 3.2 ± 1.6 (mean ± SD) years and a body weight of 17 [14; 21.5] kg (median [25%, 75% percentile]) were analysed. Duration of surgery was 3 h 49 min ± 53 min, which was accompanied by an intraoperative blood loss of 150 [90; 300] ml. This corresponded to 11.7 [5.2; 21.4] % of estimated total blood volume, ranging from 0 to 75%. A minimal haemoglobin count of 8.8 ± 1.4 g/dl was measured, which was substituted with erythrocyte concentrate (100 [0; 250] ml) in 23 patients. Body core temperature dropped from 36.0 ± 0.7°C at baseline to a minimum of 35.7 ± 0.7°C, and increased significantly (p < 0.001) thereafter to 37.1 ± 0.7°C until the end of surgery. A significant (p = 0.0003) correlation between duration of surgery and blood loss (Pearson r = 0.52) was observed. However, age, minimal body temperature or number of antiepileptic drugs seemed to have no impact on blood loss.

**Conclusion:**

Resective epilepsy surgery is a safe procedure even in the pediatric population, however it is associated with significant blood loss especially during long surgical procedures.

## Background

Epilepsy surgery has become a frequent procedure in certain neurosurgical centers [1]. Two basic techniques – which are disconnective [2,3] and resective procedures – can achieve seizure-freedom, depending on the location, size, and pathology of the epileptogenic focus. For resective methods, epileptogenic lesions are removed, which includes tailored resections, lobectomies, as well as selective amygdalohippocampectomy [4].

Irrespective of the technique chosen, epilepsy surgery has been shown to be a safe and effective treatment for medically intractable epilepsy [5]. This applies to pediatric patients as well, who represent a significant part of the patients undergoing epilepsy surgery since epilepsy often emerges in childhood [6].

Although there are several studies on safety and outcome of epilepsy surgery in general [3,7-10], only little is known about pediatric epilepsy surgery [2,11,12]. So far, only few manuscripts on the characteristics of epilepsy surgery in childhood and its implications for the anesthetist have been published [13-17].

Intraoperatively, especially children are at risk for blood loss and hypothermia, due to their low absolute blood volume and relatively high body surface area, respectively. Since hypothermia has been shown to impede coagulation, it may further aggravate blood loss [18].

Therefore, we performed a retrospective study to assess blood loss and body temperature in pediatric epilepsy surgery.

## Methods

This study was conducted in compliance with the Helsinki declaration. The responsible ethics committee (Ethikkommission an der Medizinischen Fakultät der Rheinischen Friedrich-Wilhelms-Universität Bonn, Biomedizinisches Zentrum, Sigmund-Freud-Str. 25, D-53105 Bonn, Germany) granted an exemption from requiring ethics approval for this retrospective chart analysis.

Patients were identified from the epilepsy surgery program of our neurosurgical department from the year 1989 until 2011. All had received presurgical evaluation at the Department of Epileptology, which included video EEG monitoring, MRI imaging, and neuropsychological testing if possible. We included pediatric patients with resective epilepsy surgery procedures up to the age of six years. Patients undergoing disconnective surgery and children with missing anesthesia protocols or anesthesia protocols with insufficient data were excluded.

In a retrospective chart review, we extracted data for sex, age, weight, body height, preoperative Hb and coagulation status, duration of surgery, duration of anesthesia, duration of ventilation, hours of ICU stay, blood loss, blood transfusion, administered liquids, minimal hemoglobin count, body temperature, and numbers of antiepileptic drugs. We estimated 80 ml/kg body weight total blood volume for calculation of percentage of blood loss [19,20]. Blood loss was measured as the volume within the suction container minus the volume of the irrigation fluid used. Dedicated suction containers were used for pediatric neurosurgery which allowed us to measure blood loss with an estimated accuracy of approximately 20 ml.

Data are displayed as mean ± standard deviation in case of normal distribution, or as median [25%; 75% percentile] otherwise. Normal distribution was tested using the Shapiro-Wilk-test, and relationship between variables was analysed applying Pearson’s product moment correlation. Statistical significance was assumed at a p < 0.05.

## Results

Fifty-five children with resective epilepsy surgery could be identified. Missing anesthesia protocols or charts led to the exclusion of 10 patients, leaving 45 children for final analysis. They consisted of 21 girls and 24 boys with an age of 3.2 ± 1.6 years, a body weight of 17 [14; 21.5] kg and a height of 104 [95; 113.3] cm (see Table 1 for demographic data). Children received 4 ± 2 different antiepileptic drugs, ranging from 1 to 9. One patient suffered from a Sturge-Weber-Syndrome and another two from Tuberous Sclerosis Complex. The other 42 children suffered from seizures caused by local lesions such as focal cortical dysplasia, or tumors.

**Table 1:** Demographic and surgery related data

**Parameter**		**Mean ± sd**	**Range**
Gender		21 f/24 m	
Age	[years]	3.2 ± 1.6	0 .. 6
Body weight	[kg]	17 [14; 21.5]	10 .. 32
Height	[cm]	104 [95; 113.3]	65 .. 120
Duration of			
Surgery	[hh:mm]	3:49 ± 0:53	1:40 .. 5:40
Anesthesia	[hh:mm]	5:26 ± 1:06	3:10 .. 8:45
Ventilation	[hh:mm]	6:15 [5:45; 6:55]	4:10 .. 12:45
ICU stay	[hh:mm]	19:00 [17:27; 20:00]	7:30 .. 27:00

Data are displayed as mean ± standard deviation in case of normal distribution, or as median [25%; 75% percentile] otherwise. Data were obtained from 45 children.

Preoperative values for Hb, and coagulation status could be obtained in 33 patients. Prothrombin ratio (Quick) and partial prothrombin time (PTT) were 12.6 ± 1.6 g/dl, 99.8 ± 10.7%, 27.6 ± 1.9 s, respectively.

The children received midazolam for premedication. Anesthesia was induced with intravenous thiopental at a dosage of 7.0 ± 2.4 mg thiopental per kg body weight in 26 children. 10 children received induction with Propofol. Inhalational induction with sevoflurane was performed in 9 children, in which venous access would have been difficult to achieve in the awake state. Anesthesia was maintained with isoflurane except in 4 more recent cases (2010–2011) where propofol was applied with a target-controlled infusion (TCI) pump for total intravenous anesthesia (TIVA). TIVA was guided with Bispectral Index - monitoring. Fentanyl or remifentanil were used as opioids, and vecuronium or cis-atracurium for muscle relaxation. Core temperature was monitored in all children via rectal probes All 45 patients received arterial cannulae, and 4 received a central-venous access.

Mean duration of surgery and anesthesia was 3:49 ± 0:53 h and 5:26 h ± 1:06 h, respectively. Duration of ventilation (including intensive care unit) was 6:15 [5:45; 6:55] h, which was accompanied by a high variability in length of ICU stay ranging between 7:30 h and 27:00 h (median 19:00 [17:27; 20:00] h), see Table 1 for surgery related data).

**Figure 1:**
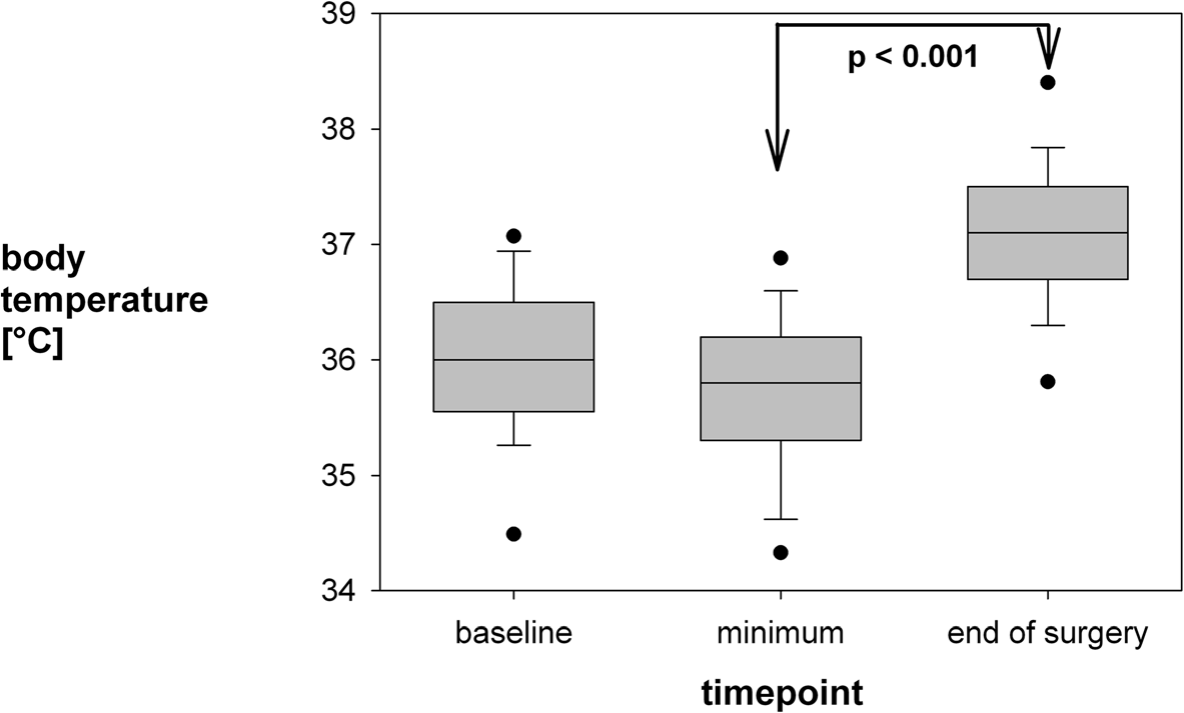
**The body temperature, as obtained during baseline (left side) and the end of surgery (right side).** In addition, the minimum intraoperative temperature is displayed in between. Body temperature increased significantly from minimum to end of surgery by forced-air warming. Data are shown as median, 25/75% and 5/95% percentiles.

**Figure 2:**
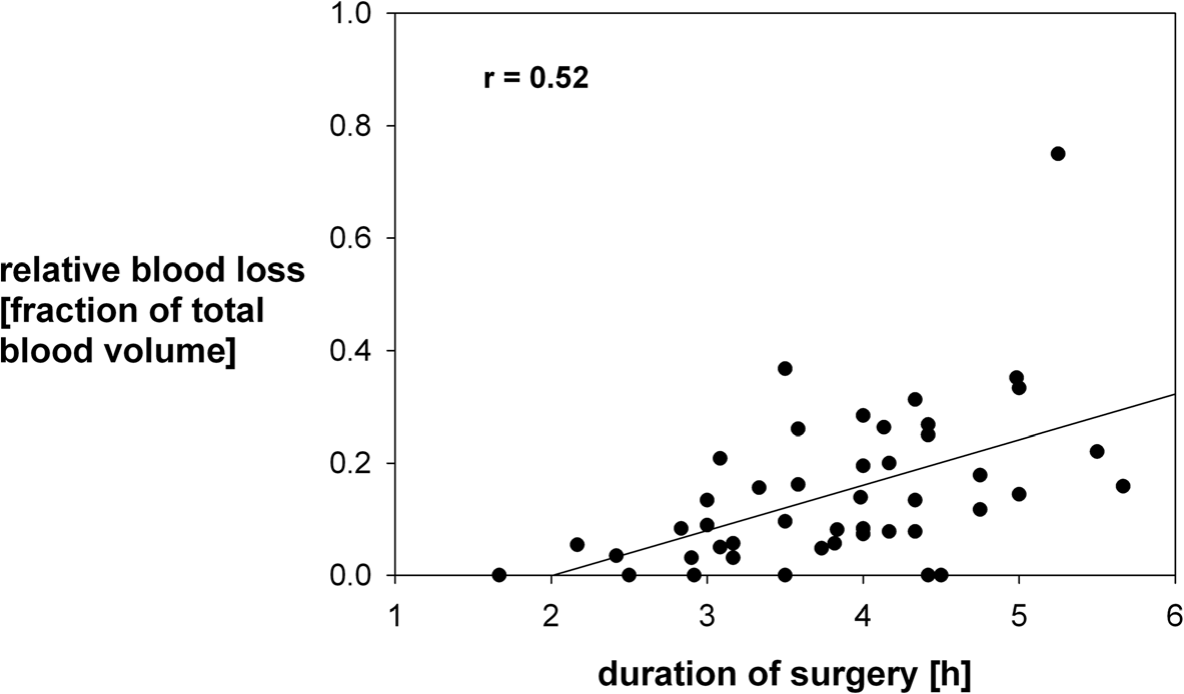
**The relation between intraoperative blood loss (expressed as fraction of the total blood volume) and the duration of surgery: a significant positive correlation (Pearson r = 0.52, p = 0.0003) was observed between the duration of surgery and blood loss.** Linear regression revealed: blood loss = 0.081 × duration of surgery – 0.163.

An absolute blood loss of 150 [90; 300] ml was observed (see Table 2), which corresponds to 11.7 [5.2; 21.4] % of total blood volume, with a high variability from 0 to 900 ml (corresponding to 0 up to 75%). This blood loss resulted in a minimal intra-operative hemoglobin count of 8.8 ± 1.4 g/dl, which was substituted with 100 [0; 250] ml red-cell transfusions in 23 patients (51%). The patient with Sturge-Weber-Syndrome and the two with Tuberous Sclerosis Complex did not differ in their blood loss from non-syndromic patients. A hemoglobin-threshhold of 9.5 g/dl was usually trigger for red-cell transfusion, although in 3 cases hemoglobin was below 9.5 and no blood was transfused. 230 to 330 ml of fresh frozen plasma (FFP) were given in 3 patients, usually whenever the estimated blood loss exceeded one third of the total blood volume. However, platelets were transfused in none of the patients. There was great variation in the amount of isotonic fluids used ranging from 250 to 1,600 ml, median 750 [500; 1000] ml. Fluids were constantly replaced during surgery at different rates. We did not observe any significant changes in heart rate or blood pressure indicating beginning hypovolaemia.Body core temperature dropped slightly from 36.0 ± 0.7°C at baseline to a minimum of 35.7 ± 0.7°C (range 34.0 .. 37.1°C), and increased significantly (p < 0.001) thereafter to 37.1 ± 0.7°C at the end of surgery (see Figure 1). The temperature nadir was reached approximately one hour after induction of anaesthesia. Rewarming was performed via a heating blanket.Pearson product moment correlation indicated a significant (p = 0.0003) correlation between duration of surgery and relative blood loss (in percent of total blood volume, Pearson r = 0.52, see Figure 2). In addition, duration of surgery correlated significantly with minimal haemoglobin concentration (r = -0.42, p = 0.006) and with the volume of erythrocyte concentrate transfusion (r = 0.50, p = 0.0005). However, no correlations were found between age, minimal body temperature, or number of antiepileptic drugs and relative blood loss. There was also no significant relationship between age and minimal body temperature.

**Table 2:** Transfusion and fluid management

**Parameter**		**Mean ± sd**	**Range**
Intraoperative blood loss	[ml]	150 [90; 300]	0 .. 900
[% of total blood volume]		11.7 [5.2; 21.4]	0 .. 75
Minimal hemoglobin concentration	[g/dl]	8.8 ± 1.4	5.7 .. 11.7
Transfusion of			
Erythrocyte concentrate	[ml]	100 [0; 250]	0 .. 770
Intravenous fluid administration	[ml]	750 [500; 1000]	250 .. 1,600

Data are shown as mean ± standard deviation in case of normal distribution, or as median [25%; 75% percentile] otherwise. n = 45 children.

No vasopressors had to be used, and no resuscitation or deaths occurred in any of the cases.

## Discussion

While there is some literature regarding neuroanesthesia and even literature dealing with pediatric epilepsy surgery, complications of standard procedures in epilepsy surgery remain largely unknown [14,15]. To our knowledge, this is the first report on blood loss and hypothermia in a relatively large cohort of children undergoing epilepsy surgery.

### Blood loss

We found that blood loss is one major factor compromising the safety of surgery, which is in accordance with Pietrini et al. who reviewed neurosurgical procedures on pediatric epilepsy patients. Whereas Pietrini et al. focused on hemispherectomies [15], we were able to show that resective epilepsy surgery is associated with considerable blood loss as well. We observed that long lasting operating times are associated with high intraoperative blood loss, which has been described by Seruya et al. for a different kind of pediatric surgery (frontoorbital advancements) [21]. Data about blood loss in general intracerebral surgery in children is scarce. In our retrospective study, we cannot discern whether this correlation was caused by surgical difficulties or anatomical anomalies, the amount of resected tissue, coagulation disorders, or a combination of these factors. Pietrini et al. stress the importance of considering coagulation disorders, especially when there is massive blood loss and the amount of blood transfusion approaches or even exceeds total blood volume [15]. In any case, a highly efficient neurosurgical strategy is necessary to avoid unnecessary loss of time, which could put the patient at risk. During the long study period of 22 years, members of the surgical team were replaced by less experienced personnel. It is possible that the experience of the surgical team may correlate with length of surgery and blood loss.

Antiepileptic medication could impede coagulation, thus putting the patient at an increased risk of intraoperative bleeding. For instance, an association between antiepileptic medication and coagulation disorders has been reported for valproate, carbamazepine, and gabapentin [22-24]. In contrast, Psaras et al. reported that under medication of valproate no increased bleeding could be observed [25]. We analysed whether the number of different antiepileptic drugs would affect intraoperative blood loss, but were unable to find any correlation which confirms earlier reports by Manohar et al. [26].

Blood transfusion strategy is a subject which is constantly under discussion [27]. Since rapid changes in blood pressure of heart rate could not be observed, we can assume that volume management with istonic fluids was adequate leaving the Hb value as the main parameter for blood replacement. Recently the threshold for transfusion in pediatric patients has become much more restrictive since it has become evident that a more liberal strategy results in worse outcomes. A transfusion-trigger of 7 g/dl has been suggested for pediatric patients in intensive care units [28]. While this can be applied to stable conditions, intra-operative conditions with massive blood loss in a short period of time may necessitate more liberal strategies in order to avoid critical anaemia and hypovolaemia. In our series, a trigger of 9.5 g/dl has usually been used even in recent operations. This reflects not only historical transfusion strategies, but also the dynamic intra-operative setting that may demand earlier blood transfusion.

As in red-cell transfusion, FFP transfusion practices remain controversial. According to recommendations, FFP should be given to correct microvascular bleeding in the presence of coagulation test disorders or to correct microvascular bleeding during massive blood transfusion [29]. However, in the pediatric setting even small blood losses can result in disseminated intravascular coagulopathy (DIC). Performing a coagulation test and defrosting of FFPs takes approximately 60 to 90 minutes (including transportation of blood sample and FFP) in our hospital. Given the fast dynamic of DIC, awaiting the coagulation test results in a situation of profound blood loss would have put the children in jeopardy. Therefore, fresh frozen plasma was given whenever the estimated blood loss exceeded one third of the total blood volume.

### Temperature management

There is evidence that hypothermia is frequent especially in small children which can have adverse effects such as surgical site infections and coagulation disorders [30]. In adults, already mild hypothermia (<36°C) has been reported to significantly increase blood loss and transfusion requirements in adults [18]. Mild hypothermia occurred in our study too, however we observed no association between body temperature and intraoperative blood loss. All children were sufficiently rewarmed until the end of surgery using forced air warming blankets, which have been shown to be effective in maintaining intraoperative hypothermia [31]. Hence, intraoperative hypothermia is of concern in children, however modern heating devices seem to have resolved this issue in the majority of patients. This seems to especially true in the neurosurgical setting, where the entire patient (except for the head) could be covered with heating blankets.

### Induction and monitoring

In our series, thiopental and, more recently, propofol were used for intravenous induction of anesthesia. We did not observe an effect of antiepileptic drugs on the dosage of thiopental for induction, which has been described to be between 5 and 8 mg/kg body weight, depending on age [32]. Propofol was administered via TCI pump for 4 recent cases. This reflects the fact that TIVA has become popular in the field of pediatric anesthesia especially when combined with monitoring depth of anesthesia [33]. Sevoflurane has become a standard agent for inhalational induction of anesthesia in children. However, excessive high sevoflurane concentrations are to be avoided due to its epileptogenic potential [34].

Intraoperative fluid management can be challenging especially in the pediatric setting, especially when there is significant blood loss that has to be restored. hence, sufficient invasive or non-invasive monitoring is necessary, and arterial cannulation is recommended in intracranial resections. In fact, all of our patients received arterial cannulae.

In contrast, the use of central venous catheters is controversial in the pediatric setting [14]. Only 4 of our patients received a central venous catheter. Since no vasopressors had to be used, it seems that fluid management was adequate without central venous catheters. However, invasive monitoring including methods such as PiCCO might be advantageous in certain major resections as it has been suggested by Pietrini et al. [15]. None of children required cardiopulmonary resuscitation, and no no case fatalities occurred. We therefore regard resective epilepsy surgery as a safe procedure even in small children.

## Conclusion

We conclude that resective epilepsy surgery is associated with a significant blood loss, especially during surgery with long operating times. This has to be taken into consideration by the anesthetist and the neurosurgeon especially in pediatric patients where fluid and blood management is more critical than in the adult population. Intraoperative hypothermia is of concern in children, however forced air warming seems to be effective in restoring normothermia in pediatric epilepsy surgery.

## Competing interests

The authors declare that they have no competing interests.

## Authors’ contributions

MT and MS designed the study and drafted the manuscript, MT, MvL, CW, and JCSB were responsible for data acquisition. All authors revised the manuscript critically for important intellectual content. All authors read and approved the final manuscript.

## Pre-publication history

The pre-publication history for this paper can be accessed here:

http://www.biomedcentral.com/1471-2253/14/71/prepub
